# Ultrasound-guided catheterization of the left subclavian vein without recognition of persistent left superior vena cava: A case report: Erratum

**DOI:** 10.1097/MD.0000000000010008

**Published:** 2018-02-16

In the article, “Ultrasound-guided catheterization of the left subclavian vein without recognition of persistent left superior vena cava: A case report”^[1]^, which appeared in Volume 96, Issue 19 of *Medicine*, the authors would like to add “This work was supported by Soonchunhyang University Research Fund.”
